# A Controlled Fermented *Samjunghwan* Herbal Formula Ameliorates Non-alcoholic Hepatosteatosis in HepG2 Cells and OLETF Rats

**DOI:** 10.3389/fphar.2018.00596

**Published:** 2018-06-19

**Authors:** AbuZar Ansari, Shambhunath Bose, Jayanta Kumar Patra, Na Rae Shin, Dong-Woo Lim, Koh-Woon Kim, Jing-Hua Wang, Young-Mi Kim, Young-Won Chin, Hojun Kim

**Affiliations:** ^1^Department of Rehabilitation Medicine of Korean Medicine, Dongguk University, Goyang, South Korea; ^2^NosQuest, USPACE 1A-1103, Seongnam, South Korea; ^3^Research Institute of Biotechnology and Medical Converged Science, Dongguk University, Goyang, South Korea; ^4^Department of Pathology, College of Korean Medicine, Dongguk University, Goyang, South Korea; ^5^Department of Korean Rehabilitation Medicine, College of Korean Medicine, Kyung Hee University, Seoul, South Korea; ^6^College of Pharmacy and Integrated Research Institute for Drug Development, Dongguk University-Seoul, Goyang, South Korea

**Keywords:** fermentation, hepatosteatosis, postbiotic, *Samjunghwan*, *Lactobacillus lactis*, *Lactobacillus bravis*, *Lactobacillus plantarum*

## Abstract

Hepatosteatosis (HS), a clinical feature of fatty liver with the excessive intracellular accumulation of triglyceride in hepatocytes, is manifested by perturbation of the maintenance of liver lipid homeostasis. *Samjunghwan* (SJH) is an herbal formula used mostly in Korean traditional medicine that is effective against a number of metabolic diseases, including obesity. Herbal drugs, enriched with numerous bioactive substances, possess health-protective benefits. Meanwhile, fermented herbal products enriched with probiotics are known to improve metabolic processes. Additionally, current lines of evidence indicate that probiotics-derived metabolites, termed as postbiotics, produce the same beneficial effects as their precursors. Herein, the anti-HS effects of 5-weeks naturally fermented SJH (FSJH) was investigated with FSJH-mixed chow diet *in vivo* using Otsuka Long-Evans Tokushima Fatty (OLETF) and Long-Evans Tokushima Otsuka (LETO) rats as animal models of HS and controls, respectively. In parallel, the anti-HS effects of postbiotic-metabolites of three bacterial strains [*Lactobacillus brevis* (LBB), *Lactococcus lactis* (LCL) and *Lactobacillus plantarum* (LBP)] isolated from FSJH were also evaluated *in vitro* using the FFAs-induced HepG2 cells. Feeding OLETF rats with FSJH-diet effectively reduced body, liver, and visceral adipose tissue (VAT) weights, produced marked hypolipidemic effects on serum and hepatic lipid parameters, decreased serum AST and ALT levels, and upregulated the HMGCOR, SREBP, and ACC, and downregulated the AMPK and LDLR gene expressions levels. Additionally, exposure of FFAs-induced HepG2 cells to postbiotic metabolic media (PMM) of bacterial strains also produced marked hypolipidemic effects on intracellular lipid contents and significantly unregulated the HMGCOR, SREBP, and ACC, and downregulated the AMPK and LDLR genes expressions levels. Overall, our results indicate that FSJH enriched with fermented metabolites could be an effective anti-HS formulation.

## Introduction

Hepatosteatosis (HS), which is generally known as fatty liver, is a disease state associated with excessive intracellular deposition of lipids [particularly triglyceride (TG) in hepatocytes (Bradbury, 2006)]. Perturbation of the balance between the TG uptake/synthesis and TG hydrolysis/secretion, which is critical to the maintenance of lipid homeostasis in the liver, could be a major factor contributing to impaired lipid metabolism (Koo, 2013). The onset and progression of HS can stem from augmented free fatty acids (FFAs) uptake and *de novo* lipogenesis for the increased TG synthesis, as well as decreased TG hydrolysis and fatty acid beta-oxidation (Koo, 2013). Free fatty acids trigger inflammation in hepatic tissue and disrupt the endoplasmic reticulum (ER), which can induce HS (Jo et al., 2013). HS can lead to non-alcoholic steatohepatitis (NASH) or non-alcoholic fatty liver disease (NAFLD), and finally cirrhosis, which distorts the architecture and impairs the physiological function of the liver (Farrell and Larter, 2006). Clinical evidence indicates that HS has a significant relationship with metabolic diseases including obesity, type 2 diabetes, and hyperlipidemia (Cusi, 2014). The prevalence of HS has been shown to be rising at an alarming rate each year, and it is currently recognized as the most common liver disease worldwide (Bellentani, 2017).

Accumulating evidence supports the usefulness of herbs for treating various human diseases, disorders, and illnesses (Luqman et al., 2014). Herbal drugs, which are enriched with numerous bioactive substances, possess immunomodulatory, anti-inflammatory, anti-diabetic, anti-infective, anti-allergic, and many other therapeutic properties and have therefore been used for thousands of years for the treatment of diseases (Hussain et al., 2016). *Samjunghwan* (SJH), a renowned herbal formula is composed of three medicinal herbs, Mori Fructus (*Morus alba* Linne, Moraceae), Lycii Radicis Cortex (*Lycium chinensis* Miller, Solanaceae), and Atractylodis Rhizoma (*Atractylodes japonica* Koidzumi, Compositae). This formulation has long been used as an anti-aging therapeutic agent (Lee and Jeong, 2006). Several studies have reported a number of beneficial effects of SJH or its fermented products, including neuroprotective and anti-oxidant activities (Kim et al., 2010; Han et al., 2014). Additionally, there is substantial evidence based on *in vitro* and *in vivo* experiments of the anti-obesity and hypolipidemic activities of SJH or its ingredients (Peng et al., 2011; Song M.Y. et al., 2013; Jiao et al., 2014). *Mori fructus*, the major constituent of SJH, has been shown to protect against obesity and related complications (Peng et al., 2011; Jiao et al., 2014), and the hypolipidemic effects of this herb are thought to be mediated through modulation of lipid homeostasis (Liu et al., 2009). We recently found that modified SJH alleviated FFAs-induced HS and that this effect was mediated through leptin signaling pathways (Lim et al., 2017).

Several lines of evidence indicate the beneficial health impact of fermented food products that contain probiotics (Heller, 2001; Hussain et al., 2016). In fermentation, bacterial plays a significant role and improves the bioactivity (Zhu et al., 2008). Probiotics are live bacteria that contribute to health benefits in the host and exert protective effects against a number of diseases (Goldin, 1998; Isolauri, 2001). We previously found that SJH fermented with probiotics (*L. plantarum*) inhibited adipogenic factors (Song M.Y. et al., 2013). This is in accordance with a number of studies showing that fermentation improves the pharmacological properties and beneficial impacts of medicinal herbs thought health-promoting and disease-preventing effects (Hussain et al., 2016). Such therapeutic enhancements have been shown to be linked with changes in secondary metabolites during fermentation through various mechanisms impacting biological activity. Recently, probiotics-derived soluble factors known as “postbiotics” have been shown to produce beneficial effects similar to those exerted by their original “patent”-live probiotics (Tsilingiri et al., 2012). Several active compounds have been characterized in postbiotics, including short chain fatty acids, polyamines, polyphosphates, proteins, and peptides, which have been shown to exert beneficial effects on a number of metabolic disorders (Ewaschuk et al., 2008; Patel and Denning, 2013; Cicenia et al., 2014). An earlier study revealed that application of suspensions of probiotics (whole components) such as heat-killed *Lactobacillus* strains alleviated alcohol-induced liver injury under both *in vitro* and *in vivo* conditions (Chuang et al., 2016). Furthermore, a recent study demonstrated the protective effects of postbiotic *Lactobacillus* strains against acetaminophen-induced hepatotoxicity in HepG2 cells (Dinić et al., 2017). Additionally, oral intake of heat-killed *Lactobacillus pentosus* exerted immunoprotective effects in elderly adults in a randomized, double-blind, placebo-controlled trial (Shinkai et al., 2013).

According to the original text of the Dongeuibogam, an encyclopedia of Oriental medical knowledge and techniques (Song et al., 2016), the recipe for SJH should be subjected to natural fermentation to exert adequate medicinal effects based on its theory of orientalism (Yin/Yang theory) (Heo, 1999). Based on the information and rationale mentioned above, we speculated that natural fermentation could improve the beneficial pharmacological properties of SJH, including hepatoprotective activities, and thereby potentiate the protective effects of this herb against HS as well. In the present study, first, we performed the basic *in vitro* dyslipidemic tests with SJH or fermented-SJH (FSJH) using FFAs-induced HepG2 cells as a model. Then we explored the *in vivo* anti-HS effects of 5-weeks FSJH upon mixing it with chow diet using Otsuka Long-Evans Tokushima fatty (OLETF), and Long-Evans Tokushima Otsuka (LETO) rats as animal models of HS and as control, respectively, in accordance with an earlier report (Song Y.S. et al., 2013). Additionally, to understand further the possible mode of action of FSJH, the anti-obesity and hypolipidemic activities of postbiotic metabolic media (PMM) of three bacterial strains (LBB, LCL, and LBP) isolated form naturally occurring FSJH were evaluated *in vitro* using FFAs-induced HepG2 cells as a model.

## Materials and Methods

### Chemicals

Dulbecco’s phosphate-buffered saline (DPBS) and Dulbecco’s Modified Eagle Medium (DMEM) obtained from Welgene (Gyeongsan-si, Gyeongsanbuk-do, South Korea). DeMan, Rogosa, and Sharpe (MRS) and M17 media were acquired from Becton, Dickinson and Company (United States). Fetal bovine serum (FBS), penicillin, and streptomycin were obtained from Gibco (Carlsbad, CA, United States). Oleic acid (OA), palmitic acid (PA), glucose, oil red o, and hematoxylin and eosin (H&E) were from Sigma-Aldrich (United States). Formalin and isopropanol were acquired from Junsei (Junsei Chemical Co., Ltd., Tokyo, Japan). RNAlater^®^ solution was bought from Ambion (Austin, TX, United States), while TRIzol^®^ reagent was acquired from Life Technologies (Carlsbad, CA, United States) and oligo-(dT) primers were procured from Invitrogen (Carlsbad, CA, United States). Additionally, cDNA RT PreMix was bought from Bioneer (Daejeon, South Korea), Light Cycler^®^ FastStart DNA Master SYBR Green was acquired from Toyobo (Osaka, Japan) and radioimmuno precipitation assay (RIPA) buffer was obtained from Biosesang (Gyeonggi-do, South Korea). In addition, Ez-cytox cell viability assay kit was bought from DoGenBio (Seoul, South Korea). A BCA protein assay kit was procured from Thermo-Scientific (Rockford, IL, United States) and a Bacterial Genomic DNA prep kit was obtained from Biofact (Daejeon, South Korea). A PowerFood^TM^ Microbial DNA Isolation Kit was bought from MoBio Laboratories Inc. (Carlsbad, CA, United States), a QIAquick PCR purification kit was acquired from QIAGEN (Valencia, CA, United States) and an Ampure bead kit was purchased from Agencourt Bioscience (Beverly, MA, United States). Moreover, a PicoGreen ds DNA assay kit was acquired from Invitrogen (Carlsbad, CA, United States), an insulin enzyme-linked immunosorbent assay (ELISA) kit was purchased from Abcam (Cambridge, United Kingdom) and TG, TC, HDL, AST, and ALT kits were procured from ASAN Pharmaceutical (Seoul, South Korea). For anesthesia, Zoletil^TM^ was obtained from Virbac (Carrros, France) and Rompun^TM^ was purchased from Bayer (Leverkusen, Germany).

### Preparation of Fermented *Samjunghwan* (FSJH)

The SJH formulation is composed of three herbal medicines: *Mori Fructus*, *Atractylodis Rhizoma*, and *Lycii Radicis*, which were purchased from the Department of Medicine, Dongguk University International Hospital (Goyang-si, Gyeonggi-do, South Korea). *Mori Fructus* juice was extracted and dried completely, while *Atractylodis rhizome* and *Lycii Radicis* were separately ground into powder form. These three preparations were mixed (20:1:1, w/w, respectively) thoroughly, then kept in a ceramic pot that was covered with a lid and stored in a room at 10°C–15°C for 5 weeks to allow natural fermentation. A small amount of sample was collected every week in 50 ml sterile Falcon tubes for further analysis. The below-described analyses of the SJH and FSJH samples were conducted by outside companies for bacterial identification (Microgen Inc., Seoul, South Korea) and bacterial community profiling by pyrosequencing (ChunLab Inc., Seoul, South Korea). Moreover, HPLC analysis of SJH and 5 weeks of FSJH extract was performed for molecular profiling by UPLC, and *in vitro* tests using FFAs-induced HepG2 cells. Finally, the SJH and the FSJH were outsourced to Feed Lab (Guri-si, Gyeonggi-do, South Korea) to prepare SJH or FSJH mixed chow diets in which 6 g of SJH or FSJH were mixed with 1 Kg of regular chow. The above dose of the herbs was selected on the basis of clinical dosage and reverse calculation according to FDA^1^ Guidance for industry. These diets were used subsequently for the animal experiments.

### Ultra Performance Liquid Chromatography (UPLC) of SJH or FSJH

The SJH or FSJH sample extracts were used for molecular profiling. The chromatographic separation of SJH or FSJH analytes was performed on an UPLC system (Waters Acquatic^TM^ UPLC, Waters Corp., Milford, MA, United States) using an ACQUATY UPLCBEHC^®^18 column (2.1 × 150 mm, 1.7 μm; Waters, Milford, MA, United States) and a UV/visible detector. All SJH and FSJH sample extracts were dissolved in DPBS at a final concentration of 10 mg/mL and filtered through a 0.22-μm-membrane syringe filter (Sartorius Amtsgericht, Gottingen, Germany). Aliquots (2 μL) of filtered SJH or FSJH were then injected into the UPLC system autosampler at 10°C. The mobile phase, which consisted of 0.1% formic acid in water (A) and 0.1% formic acid acetonitrile (B), was delivered at a flow rate of 0.3 mL/min using the following programmed gradient elution: 10% (B, v/v) isocratic for 5 min, 10→100% (B) for 20 min, 100% (B) isocratic for 2 min, 100→10% (B) for 0.5 min, and then 10% (B) isocratic for 2.5 min as post-run reconditioning. The column temperature was maintained at 40°C and the detection wavelength was 270 nm.

### Bacterial Community Profiling of SJH or FSJH by Pyrosequencing

For pyrosequencing analysis, which was conducted by ChunLab^2^ Inc., genomic DNA from unfermented SJH, and FSJH (week 1 and 5) samples was isolated using a PowerFood^TM^ Microbial DNA isolation kit according to the manufacturer’s instructions. The PCR amplification of V1–V3 sequences among nine hypervariable regions of bacterial 16S rRNA was performed using a C1000 Touch thermal cycler (BioRad, Hercules, CA, United States) to subject the samples to the following conditions: initial denaturation at 94°C for 2 min followed by 30 cycles of denaturation at 94°C for 30 s, annealing at 55°C for 30 s, and extension at 72°C for 1 min and then final extension at 72°C for 10 min. Amplicons were purified using a QIAquick PCR purification kit. Briefly, equimolar concentrations of each amplicon from different samples were pooled and purified using an Ampure bead kit. The amount of DNA in the purified samples was then quantified using a PicoGreen ds DNA assay kit. The mixed amplicons were subsequently amplified by sequencing beads using emulsion PCR (emPCR). Sequencing reactions were performed using a Roche/454 GS Junior system (454 Life Sciences, Branford, CT, United States) according to the manufacturer’s instructions. Sequences were sorted using their unique barcodes, after which low-quality reads (average quality score < 25 or read length < 300 bp) were removed. Primer sequences were then trimmed by pairwise alignment using the hmm-search program of the HMMER 3.0 package^3^. Trimmed sequences were clustered to correct for sequencing errors, and representative sequences were assigned to operational taxonomic units (OTUs; 97% identity). Taxonomic identification was performed using the EzTaxon-e database^4^ based on the highest pairwise similarity among the top five BLASTN results. Possible chimeric sequences were removed using the UCHIME^5^ software. The read numbers in each sample were normalized by random subsampling, after which the diversity indices were calculated using the Mothur^6^ program, an open source software package for processing data generated from various DNA sequencing methods, including 454 pyrosequencing UniFrac clustering. Analyses were performed using CLcommunity^TM^ (ChunLab Inc.), a standalone application developed to analyze various microbial populations.

### Isolation and Identification of Bacterial Strains of FSJH

Bacteria were isolated from FSJH extract in sterile saline (0.85% NaCl) at 5 weeks of fermentation. *Lactobacillus* sp. and *Lactococcus* sp. were cultured on MRS and M17 agar, respectively. Briefly, samples prepared from FSJH were diluted 10-fold with sterile saline, after which the resultant bacterial suspensions were spread on MRS or M17 agar plates and incubated at 37°C for 48 h. The bacterial colonies were randomly selected from the plates and transferred into 10-ml test tubes containing sterile MRS or M17 broth. The isolates were then purified by successive streaking on appropriate agar media before being subjected to identification. The isolates were stored at -80°C in sterile cryotubes containing MRS or M17 broth supplemented with 50% glycerol (v/v) and transported in frozen condition to Microgen for analysis (Seoul, South Korea). For bacterial identification, genomic DNA was extracted from the bacterial isolates using a Bacterial Genomic DNA prep kit in accordance with the manufacturer’s instructions. The 16S rRNA genes were then amplified by PCR using 27F and 1492R primers, after which they were subjected to sequencing. Sequences were then used for BLAST searches against gene sequences in the National Center for Biotechnology Information database (NCBI)^7^.

### Preparation of SJH or FSJH Extracts for HepG2 Cells Treatment

The unfermented SJH or FSJH samples were centrifuged at 5000 rpm for 10 min at 4°C and extracts were collected. The extracts were filtered twice through 0.22-μm filter (Corning Inc., Corning, New York, United States), and freeze-dried to obtain a dried product. Finally, these dried products of SJH or FSJH were dissolved in DPBS, and used for *in vitro* assays.

### Preparation of Postbiotic Metabolites Media (PMM) of Bacterial Strains for HepG2 Cell Treatment

Three strains of lactic acid bacteria (LBB, LCL, and LBP) were isolated and incubated separately for 24 h at 37°C in 5 mL of MRS broth. The revived cultures (100 μL) were then added separately to 10 mL fresh MRS broth and incubated for an additional 24 h at 37°C. The resultant bacterial media suspensions were subsequently centrifuged at 5000 rpm for 10 min at 4°C, after which dual filtration of the supernatant was conducted using a 0.22-μm filter (Corning Inc., Corning, New York, United States) to separate the PMM. To avoid the detrimental effects of acidic compounds, the pH of PMM was adjusted to ∼7.0 with 1 mM NaOH and used for *in vitro* test.

### 2,2-Diphenyl-1-picrylhydrazyl (DPPH) Free Radical Scavenging Activity

The DPPH assay was performed to determine the free radical scavenging activity of SJH, FSJH, or PMM of bacterial strains, according to our previous report (Lim et al., 2017). Briefly, 40 μl of SJH or FSJH extracts or PMM of bacterial strains were added to 760 μl ethanolic DPPH solution (0.3 mM), while an equal amount of ethanol without sample and DPPH served as control. After 30 min incubation in the dark, absorbance of the mixture was recorded at 517 nm on a microplate reader (VersaMax, Molecular Devices, United States). The DPPH free radical scavenging activity was calculated using the following formula: control (optical density)-sample (optical density)/control (optical density) × 100, and data were presented in percentage (%).

### Cultures, Cell Viability, and Treatments of HepG2 Cells

The human hepatocellular liver carcinoma cell line, HepG2, was purchased from the Korean Cell Line Bank (#88065; Seoul, South Korea). Briefly, cells were cultured in DMEM containing 10% FBS, 100 U/mL penicillin and 100 U/mL streptomycin in an incubator at 37°C under a humidified atmosphere of air containing 5% CO_2_. For cell viability analysis, HepG2 cells were seeded at a density of 5 × 10^4^ cells (100 μL) in 96-well plates, cultured for 24 h. HepG2 cells were treated with extracts of SJH or FSJH (50, 100, 200, or 400 μg/ml) or PMM (1% or 2% v/v) for 24 h under the same culturing conditions described above. Following this, cell culture media was replaced with fresh media, after which the cell viability was measured using an Ez-cytox assay kit according to manufacturer’s instructions and our previous report (Lim et al., 2017). For other experimental purposes, HepG2 cells were seeded at a density of 5 × 10^5^ cells (1 mL) in 12-well plates, and then cultured for 24 h. The cell culture media was then replaced with fresh media, after which cells were induced with a mixture of FFAs (1 mM/ml; OA:PA = 2:1 ratio) and treated with extracts of SJH or FSJH (400 μg/ml), or PMM of bacterial strains (1% v/v) for 24 h under the same culturing conditions described above and a series of experimental analyses were performed as described below.

### Oil Red o Staining of HepG2 Cells

After treatment with extracts of SJH or FSJH, or PMM of bacterial strains for 24 h, HepG2 cells were carefully washed twice with cold DPBS without disturbing the cells and subsequently fixed with 10% formalin solution for 5 min at room temperature. The cells were then washed gently with 60% isopropanol, after which they were stained with the working solution of oil red o in 60% isopropanol for 15 min (Lim et al., 2017). Next, stained cells were washed several times with cold distilled water and finally examined under an inverted microscope (DMI 6000, Leica, Jena, Germany) and images were acquired. The intracellular lipid-bound stains were re-dissolved in 100% isopropanol for 10 min, after which the resultant solution was transferred to a 96-well plate and the absorbance of the solution was read at 520 nm using a microplate reader (VersaMax, Molecular Devices, United States).

### Intracellular Lipid Content of HepG2 Cells

After exposure of cells to extracts of SJH or FSJH extracts, or PMM of bacterial strains for 24 h, cell culture media was removed carefully without disturbing the cells. The cells were gently washed with cold DPBS, lysed in RIPA buffer and subjected to homogenization. The homogenate was then centrifuged at 13,000 rpm for 30 min at 4°C to remove the insoluble materials, after which the intracellular levels of TG and TC were normalized against the total cellular protein content using a BCA protein assay kit (Lim et al., 2017).

### Analysis of Lipid-Regulating Genes in HepG2 Cells by qRT-PCR

Following treatment with PMM of bacterial strains for 24 h, cells were gently washed twice with cold DPBS and lysed in TRIzol^®^ reagent, after which the total RNA was extracted according to the manufacturer’s instructions. The amount of RNA was then determined and its purity was checked based on measurement of the optical density at 260 and 280 nm, respectively. To prepare cDNA, RNA was reverse transcribed using oligo-(dT) primers and a cDNA RT PreMix kit as per the manufacturer’s protocol. qRT-PCR was then conducted on a Light Cycler 480TM thermal cycler (Roche Applied Science, Mannheim, Germany) in a 96-well plate using a Light Cycler^®^ FastStart DNA Master SYBR Green kit following the manufacturer’s instructions. Briefly, amplification reactions were performed in a 20-μL PCR mixture containing 1 μL of cDNA, 10 pmol each of reverse and forward primers for a particular gene (listed in **Supplementary Table S1**), 10 μL of SYBR green I master mix and 8 μL of nuclease-free water. The conditions for PCR amplification reactions were as follows: initial denaturation at 95°C for 10 min followed by 45 cycles of denaturation at 95°C for 10 s, annealing at 61–63°C for 10 s and extension at 72°C for 10 s. After completion of the reaction, melting curve analysis was conducted to check the purity and specificity of the amplicon. All amplification reactions were performed in duplicate and gene expression was normalized using GAPDH as a housekeeping gene. The analyses and interpretation of the data were performed using the dedicated Light Cycler software (version 1.2, Roche Applied Science, Mannheim, Germany) provided by the instrument manufacturer. The relative gene expressions were quantified as per the standard 2^-ΔC_t_^ calculation, where *C*t denotes the crossing threshold value calculated by the software and Δ*C*t = (*C*t-target gene-*C*t-GAPDH). The primers shown in **Supplementary Table S1** were purchased form Microgen Company (Seoul, South Korea).

### Animals and Treatments

A total of 18-week-old Long-Evans Tokushima Otsuka (LETO) and Otsuka Long-Evans Tokushima Fatty (OLETF) rats were purchased from Otsuka Pharmaceutical Co. (Tokushima, Japan). The animals were housed individually in cages under a 12/12-h light-dark cycle (09:00–21:00) at a constant temperature (22 ± 2°C) and relative humidity varying from 40 to 60%. The animals were acclimatized under these conditions for 4 weeks with free access to regular chow diet (Feedlab, Guri-si, Gyeonggi-do, South Korea) and water. After the acclimatization period, OLETF rats were randomly segregated into the following three diet/treatment groups (*n* = 6) that received the different feed for 10 weeks: OLETF, regular chow diet; FSJH, FSJH-mixed chow diet; SJH group, SJH-mixed chow diet group. In addition, LETO rats serving as controls (LETO; *n* = 6) were fed regular chow diet for 10 weeks. The body weights of the animals were recorded weekly. The animal study and related ethical aspects were approved by the Institutional Animal Care and Use Committee (IACUC-2014-037) of Dongguk University. Animal experiments were performed in accordance with the Guide for the Care and Use of Laboratory Animals (Institute of Laboratory Animal Resources, Commission on Life Sciences, National Research Council, United States; National Academy Press: Washington DC, 1996).

### Oral Glucose Tolerance Test (OGTT) in Rats

The OGTT was performed 48 h prior to termination of the experimental schedule. A total of 16 h fasting-adapted rats in all groups were administered glucose prepared in autoclaved distilled water at a dose of 1 g/kg body weight by oral gavage. The levels of glucose in the sera from blood samples collected through the needle-punched tail vein of the animals were measured at four different time points (0, 60, 90, and 120 min) using an ACCU-CHEK Active system (Roche Diagnostics, Mannheim, Germany). The glucose area under cover (AUC) was calculated using the glucose level readings.

### Sample Collection

One day after the OGTT, animals were subjected to overnight (16 h) starvation with free access to water. The animals were then sacrificed under anesthesia induced by intraperitoneal injection of a mixture of Zoletil^TM^ and Rompun^TM^. Following exsanguination, blood was collected rapidly and sera were separated after clotting and centrifugation of blood at 3000 rpm for 15 min at 4°C. Collected sera were stored at -80°C for further biochemical analyses. The liver and adipose tissues (from the kidney, testis, and intestine) were promptly separated, washed with cold DPBS, dried, and weighed. Small portions of tissue samples from the liver were also excised immediately and stored at -80°C (for further lipid profiling), preserved in 10% formalin (for further histological evaluation), or submerged in RNAlater^®^ solution and snap frozen in liquid nitrogen (for quantitative gene expression). Stool samples were collected in sterile tubes and stored at -80°C for further lipid analysis.

### Fasting Insulin and Biochemical Profile in Serum, Liver, and Fecal Samples of Rats

The fasting insulin and lipid profiles in serum, liver, and fecal samples were measured by commercial kits according to the manufacturer’s instructions. Briefly, for fasting serum insulin, 100 μL of each standard and sample were incubated for 2.5 h, after which the solution was discarded and the wells were washed with 1 × wash solution (300 μl) four times. Next, 100 μL of 1 × biotinylated insulin detection antibody was added and samples were incubated for 1 h. The solution was again discarded and the wells were washed four times with 1 × wash solution. Next, 100 μL of 1 × HRP-streptavidin solution was added and incubated for 45 min, after which 100 μL of TMB one-step substrate reagent was added and incubated for 30 min. Finally, 50 μL of stop solution was added and the absorbance at 450 nm was read immediately. All steps were performed at room temperature with gentle shaking. To determine the hepatic lipid content, liver samples were washed with ice-cold DPBS and homogenized in DPBS on ice using a Vibra-Cells^TM^ ultrasonic liquid processor (Sonics & Materials, Newtown, CT, United States). The tissue homogenate was subsequently centrifuged at 12,000 rpm and 4°C for 5 min, after which the collected supernatant was used as the sample for analysis. The TG and TC contents of the samples were then determined using commercial kits according to the kit manufacturer’s instructions. Fecal lipid content was measured in accordance with Folch et al.’s (1957) method, with slight modification (Lim et al., 2017). Briefly, following collection, fecal samples (100–200 mg) were subjected to freeze-drying for 48 h and then weighed. A total of 1 ml of distilled water was subsequently added to the dried feces, mixed vigorously and homogenized. To this preparation, 2 ml of methanol and 4 ml of chloroform were added and mixed thoroughly, after which the mixture was kept at room temperature for 48 h. The bottom fraction enriched with the fecal lipids was then separated, completely dried and finally suspended in 2-isopropanol. The clear supernatant portion of the suspension was collected and served as the samples for TG and TC analysis using commercial kits as mentioned above.

### Liver Histology of Rats

Portions of the frozen or 10% formalin-fixed liver tissues were embedded in FCS22^®^ Frozen Section Media (Leica, Richmond, IL, United States) or paraffin, then sectioned at 5-μm thickness using a cryo-microtome (Leica CM1860, Leica Biosystem, Nussloch, Germany) and a rotary-microtome (Leica RM2235, Leica Biosystem, Nussloch, Germany), respectively. Sections were fixed on silicon-coated glass slides (Microslides, Muto Pure Chemical Co. Ltd., Tokyo, Japan), then stained with oil red o (for scrutinizing tissue lipid accumulation) or H&E (for evaluating liver histological architecture). Finally, the sections were examined under an inverted microscope (Leica DMI6000B, Leica Microsystems CMS GmbH, Wetzlar, Germany) and the images were captured.

### Analysis of Lipid-Regulating Genes in Rat’s Liver by qRT-PCR

Total RNA was extracted from the liver tissues stored in the aforementioned RNAlater^®^ RNA stabilization solution using TRIzol^®^ reagent. Reverse transcription of extracted RNA and subsequent quantitative gene expression analysis of a particular gene (described in **Supplementary Table S1**) were conducted following the same procedures as described above for HepG2 cells. The primers were purchased form Microgen Company (Seoul, South Korea) and are described in **Supplementary Table S1**.

### Statistical Analyses

All results are expressed as the means ± SD. Comparisons of groups were made by the one-way ANOVA using Tukey’s multiple comparison tests to access the effects of treatments. For all tests, GraphPad Prism (Version 5, GraphPad Software Inc.) software was used, and *P* < 0.05 was considered statistically significant.

## Results

### UPLC Analysis of Molecular Composition of SJH or FSJH

**FIGURE 1:**
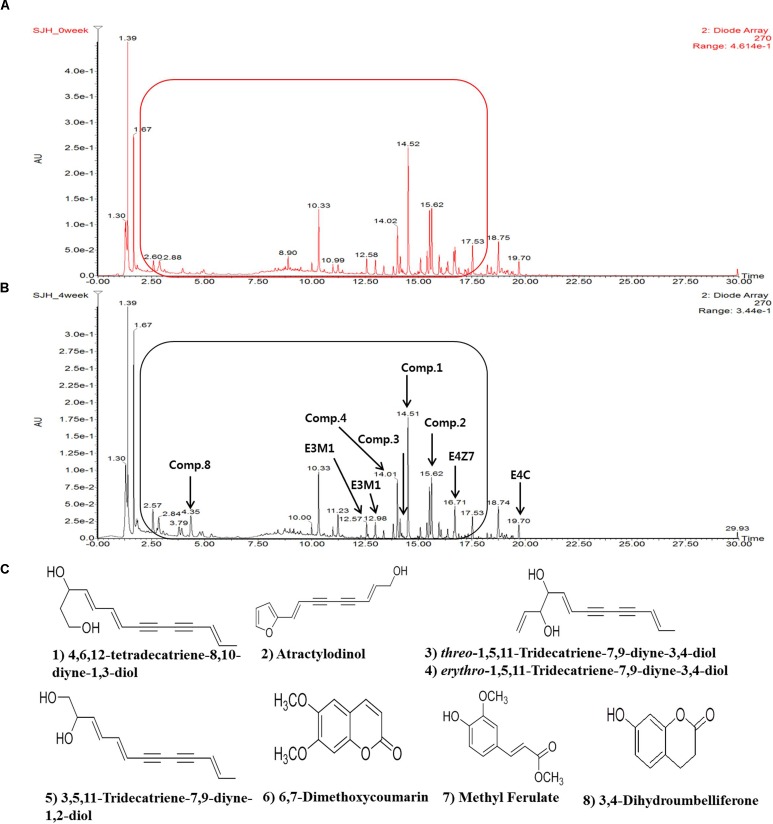
The UPLC spectra of SJH or FSJH. The comparative UPLC chromatogram of **(A)** SJH profile, **(B)** FSJH profile, and **(C)** The identified compounds indicated by arrows.

The comparative analysis of the molecular component of SJH and FSJH was performed using UPLC which revealed different peak patterns (**Figures 1A,B**). More specifically, we identified the following eight major molecules in week 5 FSJH by comparing the protonated molecular peaks appearing in UPLC chromatograms: 4,6,12-tetradecatriene-8,10-diyne-1,3-diol, atractylodinol, *Threo-*1,5,11-Tridecatriene-7,9-diyne-3,4-diol, *Erythro-*1,5,11-Tridecatriene-7,9-diyne-3,4-diol, 3,5,11-Tridecatriene-7,9-diyne-1,2-diol, Dimethoxycoumarin, methyl ferulate, 3,4-Dihydroumbelliferone (**Figure 1C**). The molecular components of the SJH components *Morus Fructus*, *Lycii Radicis* Cortex, and *Atractylodes Rhizoma* were analyzed by UPLC in our previous study (Lim et al., 2017). The identified compounds in these three plant materials were, cyanidin-3-*O*-rutinoside (Morus Fructus); betaine and kukoamine A (Lycii Radicis); Atractylodin and Atractylenolide II (Atractylodes Rhizoma).

### Fermentation-Mediated Changes in Bacterial Profile and Composition of SJH or FSJH

**FIGURE 2:**
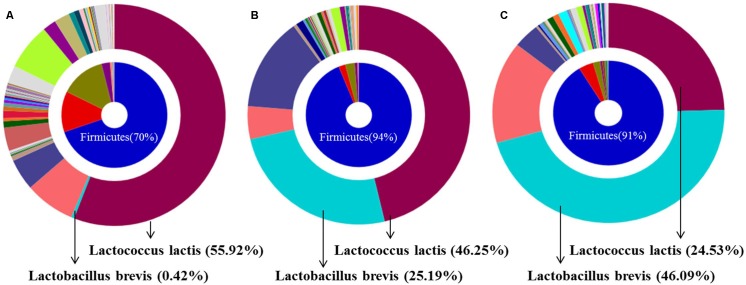
Bacterial composition of SJH or FSJH. The bacterial profile identified by pyrosequencting, **(A)** Bacterial composition of SJH (week 0), and **(B,C)** Bacterial composition of FSJH (weeks 1 and 5).

Analysis of representative microbiota by 16S rDNA bacterial pyrosequencing analysis revealed a change in the bacterial composition of SJH extract during the 5 weeks of fermentation. Specifically, the population of *L. lactis* declined gradually from week 0 (55.92%) to week 5 (24.53%), while the abundance of *L. brevis* increased dramatically from 0.42 to 46.09% during the same period (**Figures 2A,C**). This change was also observed in the week 1 FSJH compared with the week 0 SJH (**Figures 2A,B**). For more details, kindly refer to the European Nucleotide Archive^8^ (ENA) with accession no. PRJEB24751.

### Free Radical Scavenging Activity of FSJH Extract or PMM of Bacterial Strains

Prior to our *in vitro* studies, DPPH assay was performed to evaluate the antioxidant activities of SJH or FSJH extracts or PMM of bacterial strains (LBB, LCL, and LBP). The free radical scavenging activity (%) of SJH extract was found to be low, which, however, increased significantly in the course of fermentation (reflected by FSJH1 and FSJH5) (**Supplementary Figure S1A**). The PMM of bacterial strains also showed considerable free radical scavenging activity (**Supplementary Figure S1B**).

### FSJH Extract or PMM of Bacterial Strains Did Not Affect Cell Viability in HepG2 Cells

Treatment of HepG2 cells with different concentrations of SJH or FSJH extracts or PMM of bacterial strains (LBB, LCL, and LBP) for 24 h did not produce any cytotoxicity. More specifically, the cell viability of SJH or FSJH extracts or PMM of bacterial strains-treated samples at all test concentrations did not differ significantly from that of the control (**Supplementary Figures S1, S2**). Accordingly, the highest concentrations of SJH or FSJH extracts (400 μg/ml), and 1% concentration of each PMM of bacterial strains were selected for subsequent experiments.

### FSJH Extract Reduced Lipid Accumulation and Lipid Contents in HepG2 Cells

**FIGURE 3:**
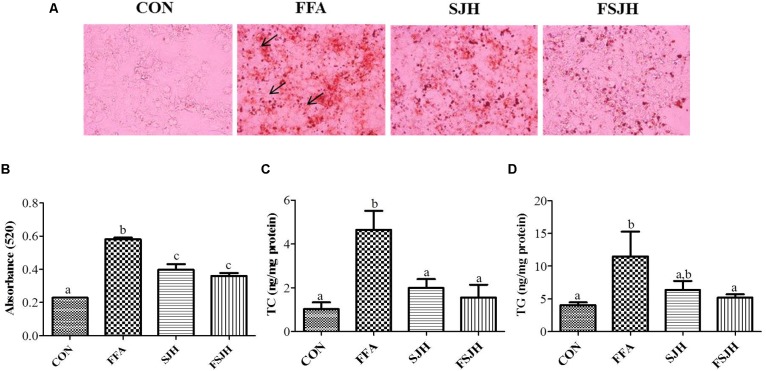
Effect of FSJH extract on lipid accumulation and intracellular lipid content in FFAs-induced HepG2 cells: **(A)** Oil red o stained images showing lipid accumulation, indicated by arrows (200× magnification and 100 μm scale bar), **(B)** Spectrophotometric-based quantification of the oil red o staining of HepG2 cells, **(C)** Total cholesterol (TC), and **(D)** Triglyceride (TG). The cells were treated with 400 μg/ml SJH or FSJH extracts. Data represent the means ± SD (*n* = 3). Statistical differences between groups were determined by one-way ANOVA. Different letters indicate statistically significant differences between groups, *P* < 0.05.

The oil red o staining was performed for lipid accumulation. Expectedly, treatment of HepG2 with FFAs for 24 h resulted in a significant increase intracellular lipid accumulation (**Figure 3A**, lipid droplets are indicated by arrow). While treatment with FSJH extract significantly decreased the lipid compared with FFAs-induced HepG2 cells, as reflected by spectrophotometric based quantitative measurement of oil red o staining (**Figure 3B**). The decreased lipid content confirmed by significant decreased TC and TG levels in FSJH extract-treated cells (**Figures 3C,D**).

### PMM of Bacterial Strains Inhibited Lipid Accumulation and Lipid Content in HepG2 Cells

**FIGURE 4:**
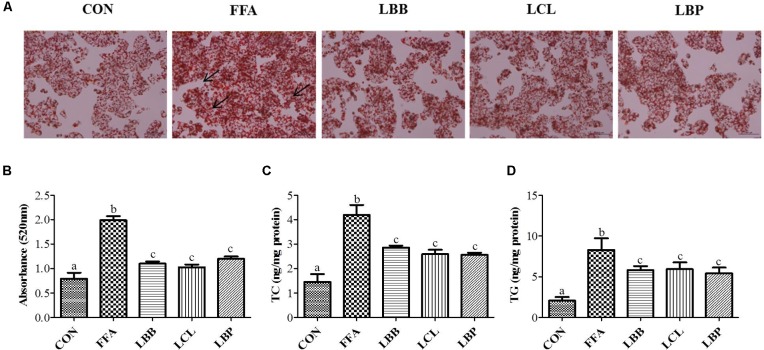
Effect of bacterial strains PMM on lipid accumulation and intracellular lipid content in FFAs-induced HepG2 cells: **(A)** Oil red o stained images showing lipid accumulation, indicated by arrows (200× magnification and 100 μm scale bar), and **(B)** Spectrophotometric-based quantification of the oil red o staining of HepG2 cells, **(C)** Total cholesterol (TC), and **(D)** Triglyceride (TG). The cells were treated with 1% of PMM of bacterial strains. Data represent the means ± SD (*n* = 4). Statistical differences between groups were determined by one-way ANOVA. Different letters indicate statistically significant differences between groups, *P* < 0.05.

Effect of PMM of bacterial strains on lipid accumulation checked with oil red o staining. The oil red o staining reveals PMM significantly decreased the lipid accumulation (**Figures 4A,B**). In keeping with our findings in oil red o staining of PMM treatment on FFAs-induced HepG2 cells significantly decreased the TC and TG levels (**Figures 4C,D**).

### PMM of Bacterial Strains Modulated Key Lipid-Regulating Genes in HepG2 Cells

**FIGURE 5:**
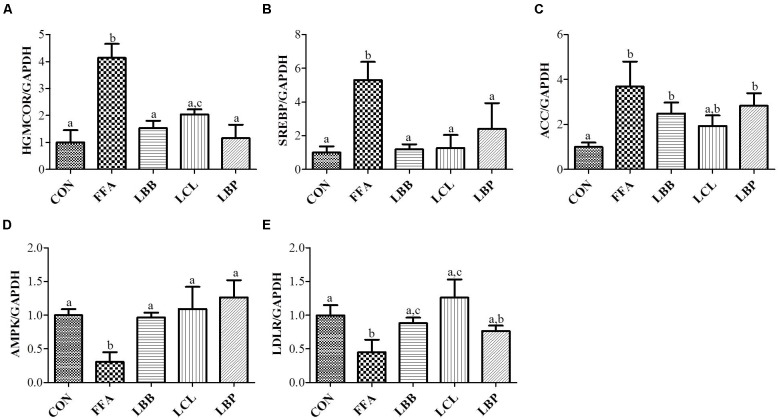
Effect of bacterial strains PMM on lipid-regulating gene expression in FFAs-induced HepG2 cells. The quantitative real-time PCR showing the impact of PMM on lipid-regulating gene expression, **(A)** HMGCOR, **(B)** SREBP, **(C)** ACC, **(D)** AMPK, and **(E)** LDLR. The gene expression of a reference protein was normalized to that of GAPDH. The cells were treated with 1% of PMM of bacterial strains. Data represent the means ± SD (*n* = 4). Statistical differences between groups were determined by one-way ANOVA. Different letters indicate statistically significant differences between groups, *P* < 0.05.

To evaluate the molecular mechanism of action of PMM of bacterial strains on the lipid metabolism in HepG2 cells, expression of vital lipid-regulating genes was analyzed by qPCR. The results revealed significantly higher expression of HMGCOR, SREBP, and ACC genes and significantly lower expression of AMPK and LDLR genes in FFAs-treated cells compared to control cells (**Figure 5**). However, exposure of FFAS-treated cells to PMM of all bacterial strains significantly decreased the expression HMGCOR and SREBP genes and significantly increased the expression of AMPK and LDLR genes. Additionally, ACC gene expression in the FFAs-treated cells was significantly downregulated when treated with PMM of LBB and LCL, but not LBP.

### FSJH-Diet Maintained Glucose Tolerance in OLETF Rats

**FIGURE 6:**
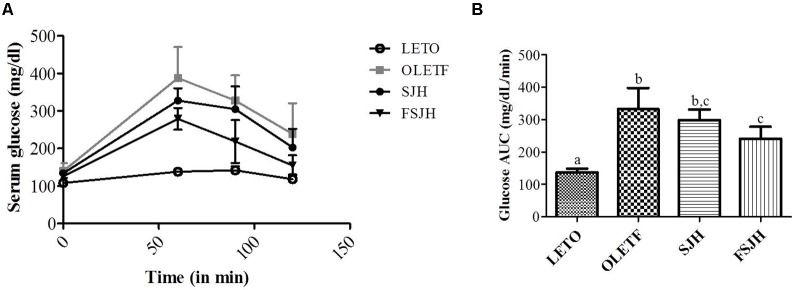
Effect of FSJH diet on glucose tolerance in rats. The oral glucose tolerance test performed on 16 h fasted rats, **(A)** Glucose level of each time points, and **(B)** Glucose AUC of glucose tolerance test. Data represent the means ± SD (*n* = 6). Statistical differences between groups were determined by one-way ANOVA. Different letters indicate statistically significant differences between groups, *P* < 0.05.

The fasting blood glucose levels were significantly higher in the OLETF group than the LETO group at every time point measured (60, 90, 120 min), while the cleaning rate of glucose in OLETF rats was significantly improved when treated with FSJH diet (**Figure 6A**) and the glucose AUC was significantly decreased compared with OLETF group (**Figure 6B**).

### FSJH-Diet Reduced Body and Liver Weights and Lipid Accumulation in OLETF Rats

**FIGURE 7:**
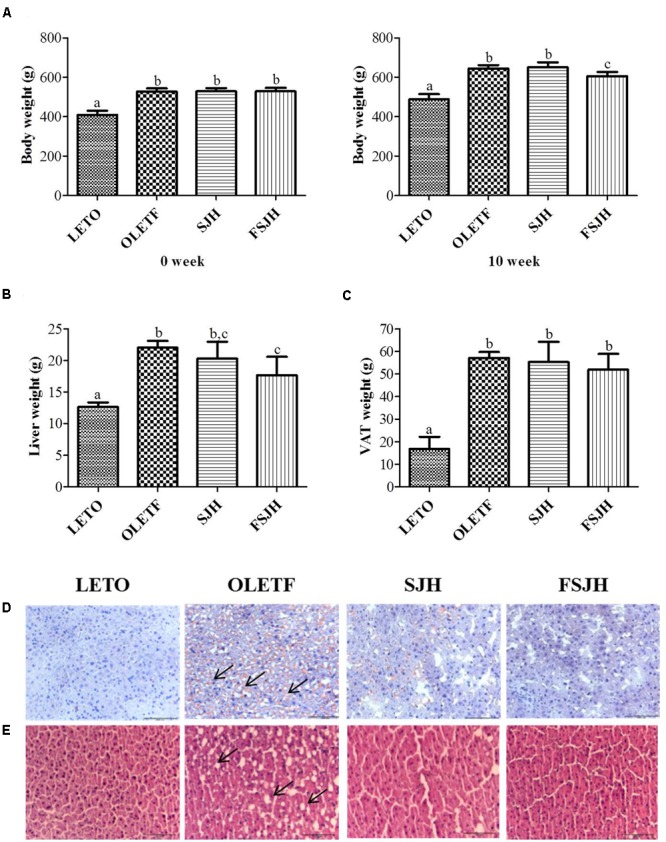
Effect of FSJH diet on the body and visceral organ weight in rats: **(A)** The comparative body weight of week 0 and week 10, **(B)** Liver weight, and **(C)** Visceral adipose tissue (VAT) during the sacrifice, **(D)** The frozen histology of liver based on oil red o staining, and **(E)** Paraffin histology based on H&E staining (200× magnification and 100 μm scale bar). Data represent the means ± SD (*n* = 6). Statistical differences between groups were determined by one-way ANOVA. Different letters indicate statistically significant differences between groups, *P* < 0.05.

Initially, there was no significant difference of body weight between groups, but the treatment of OLETF rats for 10 weeks with FSJH diet, but not SJH, effectively decreased the body and liver weights (**Figures 7A,B**). The total visceral adipose tissue (VAT) was also decreased in OLETF rats upon exposure to either SJH or FSJH-diet, but this decrease was not significant (**Figure 7C**). As expected, the oil red o and H&E staining of liver tissues of the LETO group showed no lipid accumulation and normal histological architecture without the presence of vacuoles, respectively (**Figures 7D,E**). However, high lipid accumulation (black arrows) and abnormal tissue architecture with vacuoles (black arrows) were observed in the liver tissues of the OLETF group. Markedly decreased lipid deposition and improvement in tissue architecture were evident in OLETF rats upon exposure to SJH. In contrast, OLETF rats demonstrated complete restoration of the hepatic histological structure with no trace of lipid accumulation when treated with FSJH.

### FSJH-Diet Restored the Serum Lipid Profile in OLETF Rats

**FIGURE 8:**
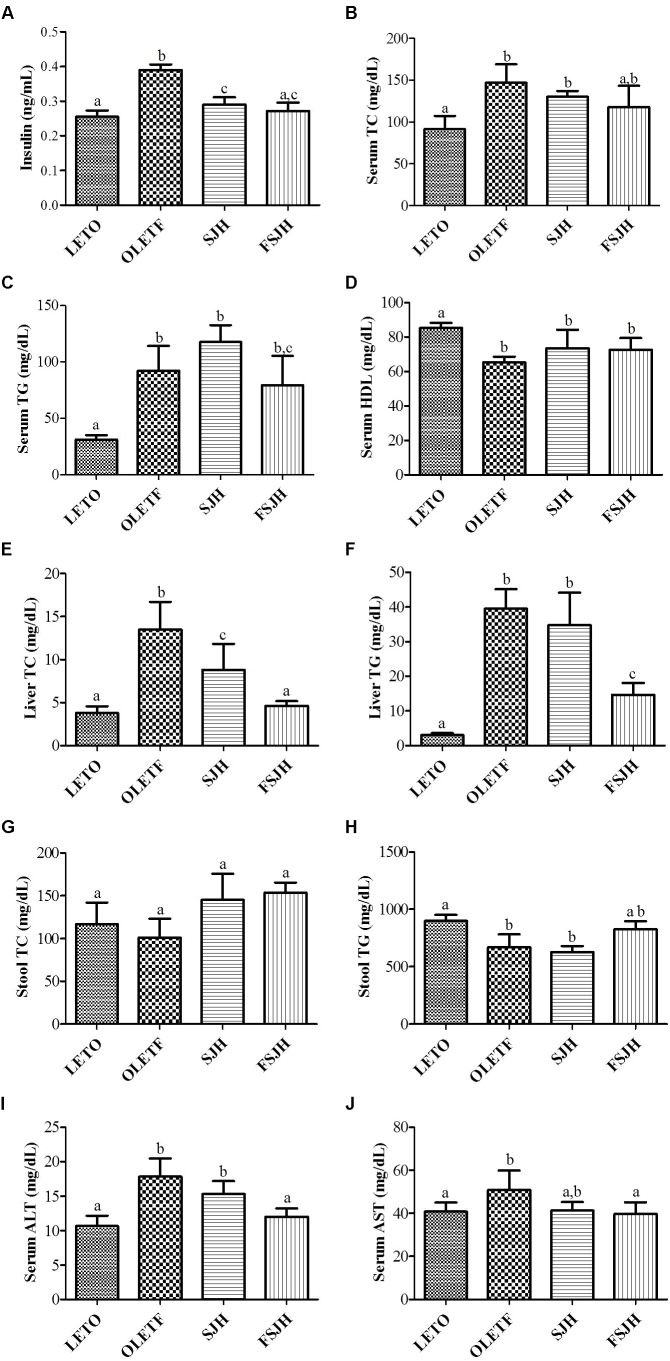
Effect of FSJH diet on serum, liver and decal biochemical-profile in rats: The serum lipid profile, **(A)** Serum insulin (INS), **(B)** Serum total cholesterol (TC), **(C)** Serum triglyceride (TG), **(D)** Serum high-density lipoprotein HDL, **(E)** Liver TC, **(F)** Liver TG, **(G)** Stool TC, **(H)** Stool TG, **(I)** Serum aspartate transaminase (ALT), and **(J)** Serum alanine transaminase (AST). Data represent the means ± SD (*n* = 3 or 6). Statistical differences between groups were determined by one-way ANOVA. Different letters indicate statistically significant differences between groups, *P* < 0.05.

The fasting insulin level of the OLETF group, which was significantly higher compared to the LETO group, decreased significantly upon exposure to both SJH and FSJH (**Figure 8A**). In the OLETF group, both the TC and TG levels were significantly higher in serum and liver and significantly lower in stool compared to those in the LETO group, while the serum HDL levels were significantly lower in the OLETF group than the LETO group. Treatment of OLETF rats with FSJH decreased the serum TC and TG levels, but this change was not significant. However, both TC and TG contents decreased significantly in the liver and increased significantly in the stool of OLETF rats upon exposure to FSJH. Treatment of the OLETF group with FSJH also increased the serum HDL level but in an insignificant manner. Notably, the gross profile of our results indicated that FSJH improved the serum, hepatic and fecal lipid parameters of OLETF rats more effectively than SJH (**Figures 8B–H**).

### FSJH-Diet Alleviated Serum AST and ALT Levels in OLETF Rats

As expected, the serum levels of both AST and ALT were significantly higher in OLETF rats compared to the LETO group(**Figures 8I,J**). However, treatment with FSJH, but not SJH, significantly decreased the serum ALT level. Exposure of OLETF rats to both SJH and FSJH reduced the serum ALT level, but this change was not significant.

### FSJH-Diet Modulated Key Lipid-Regulating Genes in OLETF Rats

**FIGURE 9:**
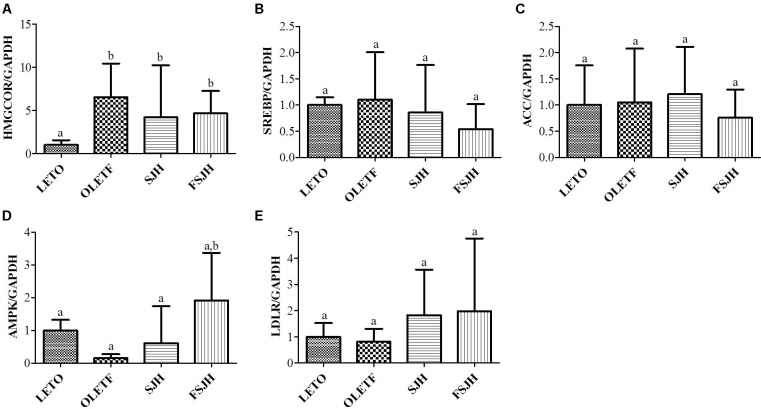
Effect of FSJH diet on lipid-regulating gene expression in the liver of rats. The quantitative real-time PCR showing the impact of FSJH diet on the lipid-metabolic genes expression, **(A)** HMGCOR, **(B)** SREBP, **(C)** ACC, **(D)** AMPK, and **(E)** LDLR. The gene expression of a reference protein was normalized to that of GAPDH. Data represent the means ± SD (*n* = 6). Statistical differences between groups were determined by one-way ANOVA. Different letters indicate statistically significant differences between groups, *P* < 0.05.

The qPCR analysis revealed that expression of HMGCOR and AMPK genes in the OLETF group was significantly higher and lower, respectively, than in the LETO group (**Figures 9A,D**), while no significant differences in expression of the SREBP, ACC, and LDLR genes were observed between OLETF and LETO rats (**Figures 9B,C,E**, respectively). Treatment of OLETF rats with FSJH, but not SJH, significantly unregulated expression of the AMPK gene. Additionally, exposure of the OLETF group to both SJH and FSJH decreased the expression of HMGCOR, SREBP, and ACC genes and increased the expression of LDLR genes, but these changes were not significant, while treatment of OLETF rats with FSJH, but not SJH, significantly upregulated expression of the AMPK gene.

## Discussion

Clinical reports have suggested that HS has a significant relationship with metabolic diseases including obesity, hyperlipidemia, and type 2 diabetes (Farrell and Larter, 2006), while a number of studies have revealed the hepatoprotective effects of herbal medicines against HS and NAFLD (Park et al., 2015). Moreover, there is growing evidence supporting the anti-obesity properties of SJH (Song M.Y. et al., 2013), an herbal formulation that has long been used for the treatment of senescent diseases (Lim et al., 2017). The composition of SJH is based on the fundamental Oriental philosophy of correspondence between nature and humans. Specifically, the components of this formulation were introduced as the essence of the sky (*Atractylodis Rhizoma*), the essence of the earth (*Lycii Radicis Cortex*), and the essence of humans (*Mori Fructus*) (Heo, 1999). This herbal formulation has been found to inhibit food intake, reduce the liver and epididymal adipose tissue weight, and inhibit the activities of hepatic acetyl-CoA carboxylase and fatty acid synthetase in rats fed high-fat diet (Jeong et al., 2006). We recently demonstrated that a modified formulation of SJH alleviates FFAs-induced HS through leptin signaling pathways (Lim et al., 2017). Accumulating evidence indicates that fermentation is an effective method for enhancing bioactivity of several herbal medicines (Hussain et al., 2016). More specifically, fermentation promotes decomposition and/or biotransformation of complex substrates into compatible components, thereby modulating the composition and chemical properties of herbs through production and enrichment of a variety of bioactive compounds of with medicinal importance, including isoflavones, saponins, phytosterols, and phenols (Hussain et al., 2016). In agreement with these findings, current lines of evidence indicate that fermentation mediates enhancement of the pharmacological properties and therapeutic efficacies of herbal formulations against a number of diseases, such as obesity and inflammation (Hussain et al., 2016). Moreover, lactic acid bacteria-mediated natural fermentation has been found to enhance total polyphenol and flavonoid contents and DPPH free radical scavenging activity of SJH (Chang et al., 2016). The measurement of free radical DPPH is one of the currently popular methods for estimating antioxidant activity in extracts (Molyneux, 2004). Here, we observer a minimal free radical scavenging activity of SJH which was markedly improved after 5-weeks fermentation of this herbal formula. Bacteria play a major role in fermentation, which improves the bioactivity including antioxidant activity (Zhu et al., 2008). In a previous report, different strains of *L plantarum* exhibited antioxidant (Li et al., 2012). In the present study, we also found the DPPH free radical activities of PMM of selected bacterial strains. Furthermore, FSJH was shown to protect HepG2 liver cells from hydrogen peroxide insult and exert anti-obesity effects in rats fed high-fat diet (Song M. et al., 2013; Chang et al., 2016).

These findings prompted us to thoroughly investigate the impact of fermentation on the hepatoprotective activity of SJH against HS and the probable underlying mechanism(s). In the present study, the anti-HS effect of FSJH was extensively examined through a series of *in vitro* and *in vivo* studies. HepG2 cells used as an *in vitro* model in this study were induced by a mixture of oleic and palmitic acids at a ratio of 2:1 (w/w) in accordance with our previous report (Lim et al., 2017). These two fatty acids account for the majority of the hepatic triglyceride pool in both normal subjects and volunteers suffering from NAFLD (Yang et al., 2000). Furthermore, exposure of HepG2 cells to a combination of oleic and palmitic acids at a certain ratio triggers abundant intracellular lipid accumulation (Lim et al., 2017), resulting in a triglyceride content comparable to that measured in the livers of humans suffering from steatosis (Gómez-Lechón et al., 2007). Excess FFAs accumulation in the liver has also been shown to favor HS, and evidence suggests that FFAs are important to obesity, insulin resistance, and type 2 diabetes (Fabbrini et al., 2010; Zhang et al., 2014). In the present study, OLETF and LETO rats were used as animal models of HS and as controls, respectively, in accordance with a previous report (Song Y.S. et al., 2013). OLETF rats have been found to develop HS spontaneously without the need for extreme dietary manipulation (Kurita et al., 2008), and the natural progression of this disease closely resembles that occurring in obese humans (Rector et al., 2010).

In agreement with the above findings, oil red o staining in the present study showed that exposure of HepG2 cells to a mixture of oleic and palmitic acids (FFAs) at a ratio of 2:1 (w/w) for 24 h caused a significant increase in intracellular fat accumulation. Further biochemical analysis revealed that intracellular levels of TC and TG levels were significantly augmented, while those of HDL were significantly decreased in cells treated with FFAs. As a representative model of NAFLD (Rector et al., 2010), OLETF rats typically feature elevated hepatic TG levels and hepatocyte ballooning. In keeping with this, we found the aberrant histological architecture of the liver with the substantial accumulation of fat in the OLETF rats. This was further supported by biochemical studies demonstrating significantly higher hepatic levels of TC and TG and elevated serum levels AST and ALT in OLETF rats compared to the LETO group. In parallel, significantly higher visceral adipose tissue weight and serum levels of TG and TC accompanied by significantly lower serum HDL concentrations were observed in the OLEFT group compared to the LETO group. Fat accumulation in the body and in the liver is closely associated with body fat mass, glucose and insulin metabolism (Seppälä-Lindroos et al., 2002). In our oral glucose tolerance test, the fasting blood glucose levels were found to be significantly higher in OLETF rats compared to the LETO group at every time point evaluated. Moreover, the fasting insulin level of the OLETF group was significantly higher than that of LETO rats. Obesity and dyslipidemia are considered to be the major factors contributing to the onset and development of HS, and hepatic lipid accumulation and insulin resistance are proposed to be the key factors contributing to steatosis development (Day, 2002).

**FIGURE 10:**
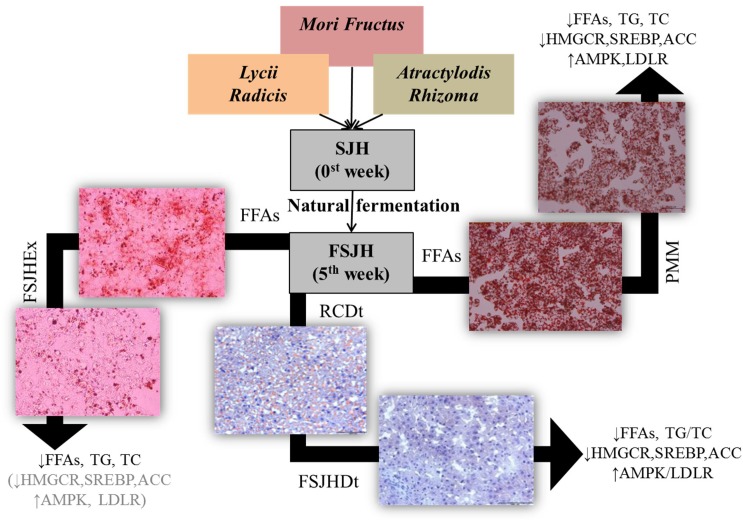
Effect of FSJH or PMM on hepatosteatosis. Impact of FSJH extract or diet or PMM of bacterial strains on the lipid accumulation, lipid contents, and lipid-regulated genes expression. SJH = *Samjunghwan*, FSJH = Fermented SJH, FFAs = Free Fatty Acids, FSJHEx = FSJH Extract, PMM = Postbiotic metbolic media of bacterial strains, RCDt = Regular Chow Diet, and FSJHDt = Fermented *Samjunghwan* Diet.

The existing options for treatment of HS mainly rely on weight loss through maintenance of diet and exercise (Hickman et al., 2004; Huang et al., 2005). In addition, hepato-protective agents such as anti-oxidants, anti-inflammatory drugs, and insulin sensitizing medicines are regularly prescribed to protect the liver from further damage (Merat et al., 2003; Schwimmer et al., 2005; Masterton et al., 2010). Notably, several lines of evidence have indicated that treatment with a number of herbal formulations, including SJH, is an effective strategy for obesity control and weight management (Hussain et al., 2016). Moreover, because of their appreciable hepatoprotective activities, several herbal medicines have become increasingly popular as alternative options for the treatment of liver diseases (Hussain et al., 2016). Emerging evidence also suggests the beneficial effects of fermentation on the anti-obesity and hepatoprotective activities of herbal drugs (Hussain et al., 2016). The compositional change in molecular components and bacterial profile of SJH to FSJH were observed. Here, we found after fermentation, the metabolic actability SJH was improved markedly, most likely through enrichment of its fermented metabolites and postbiotic metabolites of bacterial strains involved in fermentation. The anti-HS effect of FSJH was further supported by our *in vitro* and *in vivo* experiments. In *in vitro* study, treatments of FFAs-induced HepG2 cells with FSJH-extract or PMM of bacterial strains decreased the accumulation of FFAs and modulated the lipid-regulating genes. Similarly, in our *in vivo* study, feeding OLETF rats FSJH-diet reduced lipid accumulation and influenced the lipid-regulating genes (**Figure 10** and Supplementary Table S2).

In agreement with the above, our overall findings in the animal studies revealed appreciable anti-obesity effects, as well as significant lipid excretion and hepatic lipid-lowering effects of FSJH. Specifically, treatment of OLETF rats with FSJH significantly reduced body and liver weights, improved hepatic histological architecture, inhibited lipid accumulation, and reduced TC and TG levels in the liver. In parallel, the levels of fecal TC and TG were also significantly elevated. Moreover, the serum levels of AST and ALT in OLETF rats were increased significantly and markedly, respectively, upon exposure to FSJH, further supporting the hepatoprotective activity of this herbal formulation. Moreover, in our study, the evaluation of hepatic gene expression using real-time PCR revealed that the FSJH treatment regulates the expression of vital genes influencing lipid metabolism. In keeping with this, although insignificantly, but a definite reduction in the hepatic expression of HMGCOR, the principal enzyme of cholesterol biosynthesis which offers a promising approach for the treatment of hypercholesterolemia (Grundy, 1988) was observed in OLETF rats due to exposure to FSJH. AMPK is an energy sensor that regulates cellular metabolism including lipid signaling (Long and Zierath, 2006). Activated AMPK prevents lipid deposition in muscle and liver (Zhou et al., 2001; Collier et al., 2006). AMPK activation triggers the suppress expression of ACC via downregulation of SREBP, attenuate hepatic steatosis (Li et al., 2011). In our study, FSJH significantly increased the expression of AMPK and although insignificantly, but caused a definite reduction in the expression of ACC and SREBP in OLETF rats. Additionally, the gene expression LDLR, a key player in the cholesterol transport (Brown and Goldstein, 1986), was improved by FSJH treatment in OLETF rats.

Probiotics and their fermented herbal products are known to exert beneficial impacts on the health of hosts (Hussain et al., 2016). Several lines of evidence indicate the anti-obesity and hepatoprotective activities of probiotics. Moreover, probiotics supplements have been reported to regulate abdominal adiposity and attenuate liver fat accumulation, and shown to be effective in the treatment of NAFLD (Kadooka et al., 2010; Xu et al., 2011). In a study by (Li et al., 2003), ob/ob mice fed a diet containing VSL#3, a combination of probiotic bacteria, for 4 weeks showed marked reduction in inflammation, total fatty acid content and fatty acid β*-*oxidation of the liver, as well as a significant reduction in serum ALT levels accompanied by improved hepatic insulin resistance. Similarly, Proxetin, another formulation of probiotics, was found to effectively reduce the serum ALT and AST levels in patients suffering from NAFLD (Eslamparast et al., 2014). Both *L. brevis* and *L. lactis*, the two most abundant bacterial species found in our FSJH preparation, are lactic acid bacteria, which are known to include many species with probiotic properties (Ljungh and Wadstrom, 2006). Additionally, several lines of evidence indicate the strain-specific probiotic activities of *L. lactis* (Yadav et al., 2009; Han et al., 2015; Lee et al., 2015; Armas et al., 2017; Oliveira et al., 2017) and *L. brevis* (Rönkä et al., 2003; Linsalata et al., 2004; Fukao et al., 2013; Waki et al., 2014). Moreover, for the past few years, a large number of studies have revealed the probiotic health effects of *L. plantarum* (Seddik et al., 2017). Indeed, *L. plantarum* strains were shown to be potential probiotic cultures with cholesterol-lowering activity (Huang et al., 2013), and reduced serum lipid parameters (TC and TC levels) were observed in Wistar rats exposed to a native strain of *L. plantarum* A7 (Fazeli et al., 2010).

Accumulating evidence has revealed that probiotics combat diseases through a number of mechanisms, among which their biologically active metabolic by-products and secretory molecules, collectively termed “postbiotics,” play important roles (Nguyen et al., 2007). To further elucidate the contribution of the most abundant microbial population of FSJH to the anti-HS effects of this herbal formulation, we separately evaluated the anti-hyperlipidemic and hepatoprotective effects of the PMM of *L. lactis* and *L. brevis* using FFAs-induced HepG2 cells as a model. Moreover, for a better comparison, PMM of *L. plantarum* was used as a reference as this probiotic produces beneficial health effects and is one of the bacterial species most frequently found or employed for vegetable and fruit fermentation (Filannino et al., 2014; Seddik et al., 2017). Our overall findings revealed an appreciable anti-hyperlipidemic impact of PMM of both LBB and LCL on FFAs-induced HepG2 cells. More specifically, PMM of these two species significantly inhibited lipid accumulation and decreased TG and TC levels, while increasing HDL levels in FFAs-treated cells. Additionally, treatment of FFAs-induced cells with PMM of both LBB and LCL significantly attenuated the expression of HMGCOR, SREBP, and ACC genes and significantly upregulated the expression of AMPK and LDLR genes. These effects of PMM of both bacteria were quite comparable to the impact of PMM of LBP.

Growing evidence indicates that a variety of metabolites are derived from the microbial community. For example, several genes associated with specific metabolic pathways such as amino acid and glycan metabolism were shown to be overrepresented in the microbiota of the distal gut (Gill et al., 2006; Wikoff et al., 2009). Additionally, obese mice were found to more effectively harvest energy from their food than their lean counterparts because of changes in the composition of their gut microflora that resulted in an increased complement of genes involved in polysaccharide metabolism (Turnbaugh et al., 2006; Wikoff et al., 2009). Moreover, genes expressing bile salt hydrolase enzymes were found to be enriched in the gut microbial population, and enteric bacteria were shown to execute a wide range of bile acid modifications (Ridlon et al., 2006; Jones et al., 2008; Wikoff et al., 2009). Taking the above information together, it is conceivable that the metabolites derived from this diverse microbial community play a crucial role in human health and disease (Wikoff et al., 2009; Zhang and Davies, 2016). Investigations have revealed that these metabolites include short-chain fatty acids and long-chain fatty acid metabolites such as conjugated linoleic acid and 10-hydroxy-cis-12-octadecenoate, as well as trimethylamine and trimethylamine N-oxide, tryptophan metabolites such as indole, indole-3-propionate and indoxyl-sulfate, and tyrosine and phenylalanine metabolites such as hippuric acid, phenylacetyl glycine, phenyl sulfate, paracresyl sulfate (PCS), phenylpropionylglycine, cinnamoylglycine, and equol sulfate (Wikoff et al., 2009; Zhang and Davies, 2016).

In the present study, UPLC-MS analysis of FSJH revealed the presence of a number of compounds. Among these, 4,6,12-tetradecatriene-8,10-diyne-1,3-diol, a glycol compound with two hydroxyl residues, closely resembles the structure of 4,6,12-tetradecatriene-8,10-diyne-1,3-14 triol. The latter compound has been found in the Japanese traditional herbal medicine boiogito (BOT), which has long been used to treat patients suffering from obesity of the “asthenic constitution” type, also known as “watery obesity” (Shimada et al., 2011). The effects of this medicine have already been recognized clinically. Indeed, BOT has been shown to play an important role in hepatic TG metabolism and to prevent sodium cholate-induced liver injury in mice (Watanabe et al., 2016). Moreover, components of BOT have been shown to suppress the progression of hypercholesterolemia and fatty liver triggered by high-cholesterol diet in rats (Qian et al., 2016). Our UPLC-MS analysis also demonstrated the presence of threo- and erythro- forms of a 1,5,11-Tridecatriene-7,9-diyne-3,4-diol compound in FSJH. The derivative of this compound has also been identified in *Atractylodes lancea*, a Chinese traditional herbal medicine showing pancreatic lipase inhibitory activity and a moderate anti-obesity efficacy in HFD-induced obesity mice (Jiao et al., 2014).

The atractylodinol identified in FSJH extract is present in a number of traditional medicines. This compound has been found in BOT, which exhibits an anti-obesity effect in the Tsumura Suzuki Obese Diabetes (TSOD) mouse by suppressing body weight gain and accumulation of subcutaneous and visceral fat (Shimada et al., 2011). Atractylodinol has also been identified in the aforementioned *A. lancea* herbal drug (Jiao et al., 2014), while, 6,7-dimethoxycoumarin (scoparone) and 3,4-dihydroumbelliferone found in FSJH extract are coumarin derivatives. Both of these compounds are antioxidants and have been shown to protect the liver from carbon tetrachloride-induced injury in rats (Tanaka et al., 2017). Such hepatoprotective activity of scoparone is reportedly associated with regulated expression of six closely related proteins in the protein-protein interaction network and appeared to be involved in antioxidation and signal transduction, energy production, immunity, metabolism, and chaperon pathways (Zhang et al., 2013). In mice, scoparone has been shown to facilitate bilirubin clearance, which is accompanied by an upregulation of the expression of UGT1A1 and MRP2 genes (Huang et al., 2004). Methyl ferulate found in FSJH extract has been reported to facilitate the growth of *Lactobacillus* strains as a carbon source in the presence of phenolic acids and their derivatives (Szwajgier and Jakubczyk, 2010; Waśko et al., 2014).

## Conclusion

Probiotics can modulate the gut microflora and influence the gut-liver axis, and it is possible that these bacterial strains might participate in the lipid-metabolism process. Lactic acid bacteria isolated from FSJH have the potential to effectively reduce lipid accumulation through lipid synthesis and lipid-regulating gene expression. The FSJH have the potential to effectively control the body and liver weight in rats, as well as the expression of lipid-regulating genes. Overall, FSJH markedly attenuated HS through fermented metabolites of FSJH and postbiotic, demonstrating that these naturally fermented products can be considered anti-HS compounds to treat HS in the future.

## Author Contributions

AA wrote the manuscript and performed the *in vivo* experiment. SB and JP rewrote the manuscript. NS and D-WL performed the fermentation and *in vitro* experiments. K-WK and J-HW analyzed the data. Y-MK performed the HPLC analysis. Y-WC and HK perceived the study and managed the experiment process.

## Conflict of Interest Statement

The authors declare that the research was conducted in the absence of any commercial or financial relationships that could be construed as a potential conflict of interest.

## Footnotes

1 https://www.fda.gov/downloads/drugs/guidances/ucm078932.pdf

2 http://www.chunlab.com/

3 http://hmmer.org

4 http://eztaxon-e.ezbiocloud.net/

5 https://drive5.com

6 https://www.mothur.org

7 https://blast.ncbi.nlm.nih.gov/Blast.cgi

8 https://www.ebi.ac.uk/ena

## Supplementary Material

FIGURE S1 Effect of herbal extracts or bacterial strains PMM on free radical scavenging activity: DPPH activity of herbal extracts **(A)** SJH or FSJH, **(B)** PMM of bacterial strains (LBB, LCL, or LBP). FSJH1 = week 1 and FSJH5 = week 5 of fermentation. Data represent the means ± SD (*n* = 3). Statistical differences between groups were determined by one-way ANOVA. Different letters indicate statistically significant differences between groups, *P* < 0.05.

FIGURE S2 Effect of herbal extracts or bacterial strains PMM on cell-viability in HepG2 cells: Cell viability was determined by Ez-cytox assay. The cell viability of HepG2 cells treated with **(A)** SJH or FSJH extracts (in μg/ml), **(B)** PMM of bacterial strains (LBB, LCL, or LBP) vs. untreated controls. MRS treatment was considered a positive control in PMM treatment. Data represent the means ± SD (*n* = 6). Statistical differences between groups were determined by one-way ANOVA. Different letters indicate statistically significant differences between groups, *P* < 0.05.

TABLE S1 Sequences of primers used for qPCR. H = Human, R = Rat.

TABLE S2 Comparative analysis of herbal extract and postbiotic metabolic media of bacterial strains treatment on FFAs-induced HepG2 cells. ORO, oil red o; TC, total cholesterol; TG, triglycerides.

## ABBREVIATIONS

ACC: acetyl-CoA carboxylase
ALT: alanine transaminase
AMPK: 5^′^-AMP-activated protein kinase catalytic
AST: aspartate transaminase
FFAs: free-fatty acids
FSJH: fermented SJH
GAPDH: glyceraldehyde-3-phosphate dehydrogenase
HDL: high-density lipoprotein
HMGCOR: 3-hydroxy-3-methylglutaryl-coenzyme A reductase
LBB: *Lactobacillus brevis*
LBP: *Lactobacillus plantarum*
LCL: *Lactococcus lactis*
LDLR: low-density lipoprotein receptor
LETO: Long-Evans Tokushima Otsuka
OLETF: Otsuka Long-Evans Tokushima fatty
ORO: oil red o
PMM: Postbiotic Metabolic Media
SJH: *Samjunghwan*
SREBP: sterol regulatory element-binding protein
TC: total cholesterol
TG: triglycerides
VAT: visceral adipose tissue
