# Supplementary figures and images for: A Controlled Fermented Samjunghwan Herbal Formula Ameliorates Non-alcoholic Hepatosteatosis in HepG2 Cells and OLETF Rats
Source: Front Pharmacol. 2018 Jun 19;9:596. doi: 10.3389/fphar.2018.00596 (PMC6018163; doi:10.3389/fphar.2018.00596)

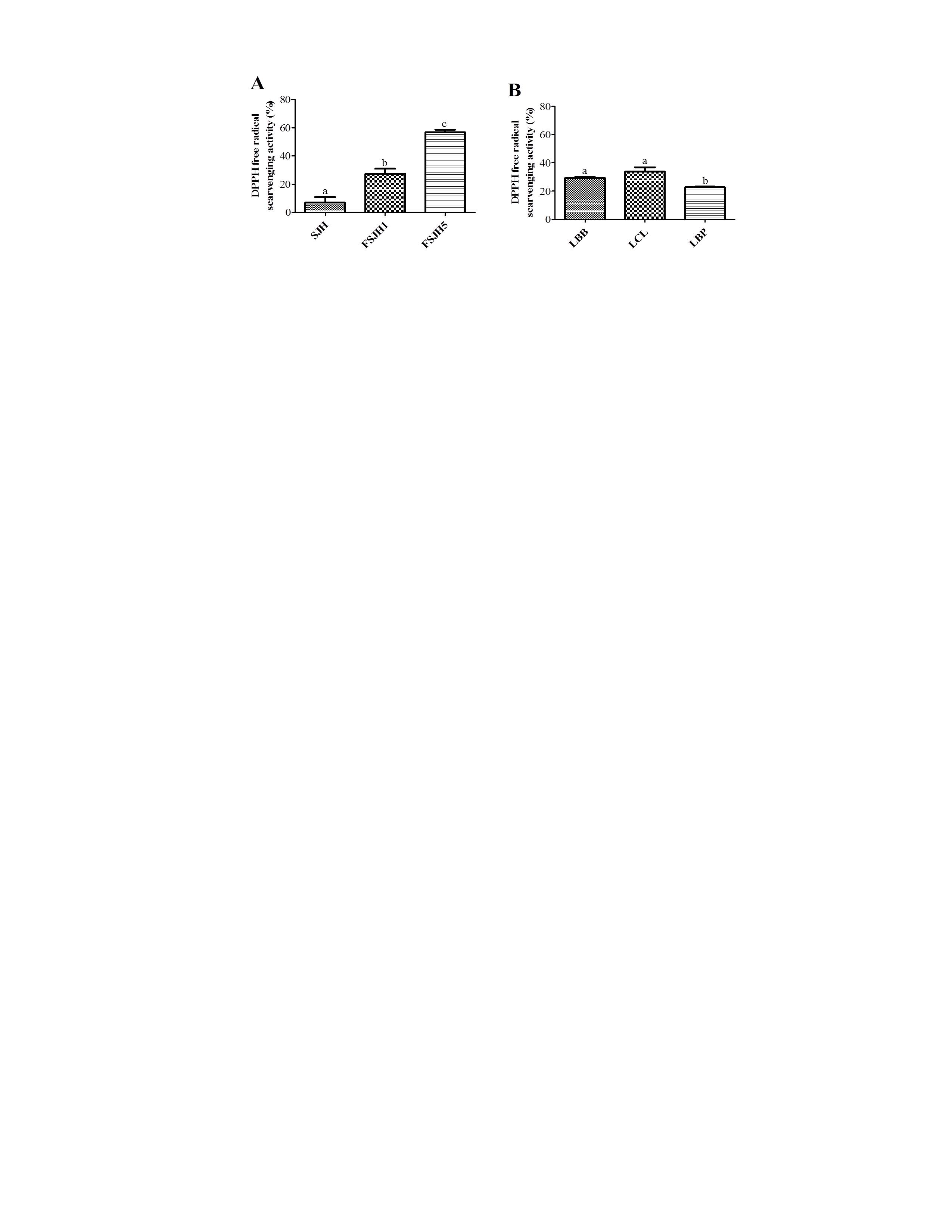

Supplement: FIGURE S1 — Effect of herbal extracts or bacterial strains PMM on free radical scavenging activity: DPPH activity of herbal extracts (A) SJH or FSJH, (B) PMM of bacterial strains (LBB, LCL, or LBP). FSJH1 = week 1 and FSJH5 = week 5 of fermentation. Data represent the means ± SD (n = 3). Statistical differences between groups were determined by one-way ANOVA. Different letters indicate statistically significant differences between groups, P < 0.05. [file Image_1.jpg]

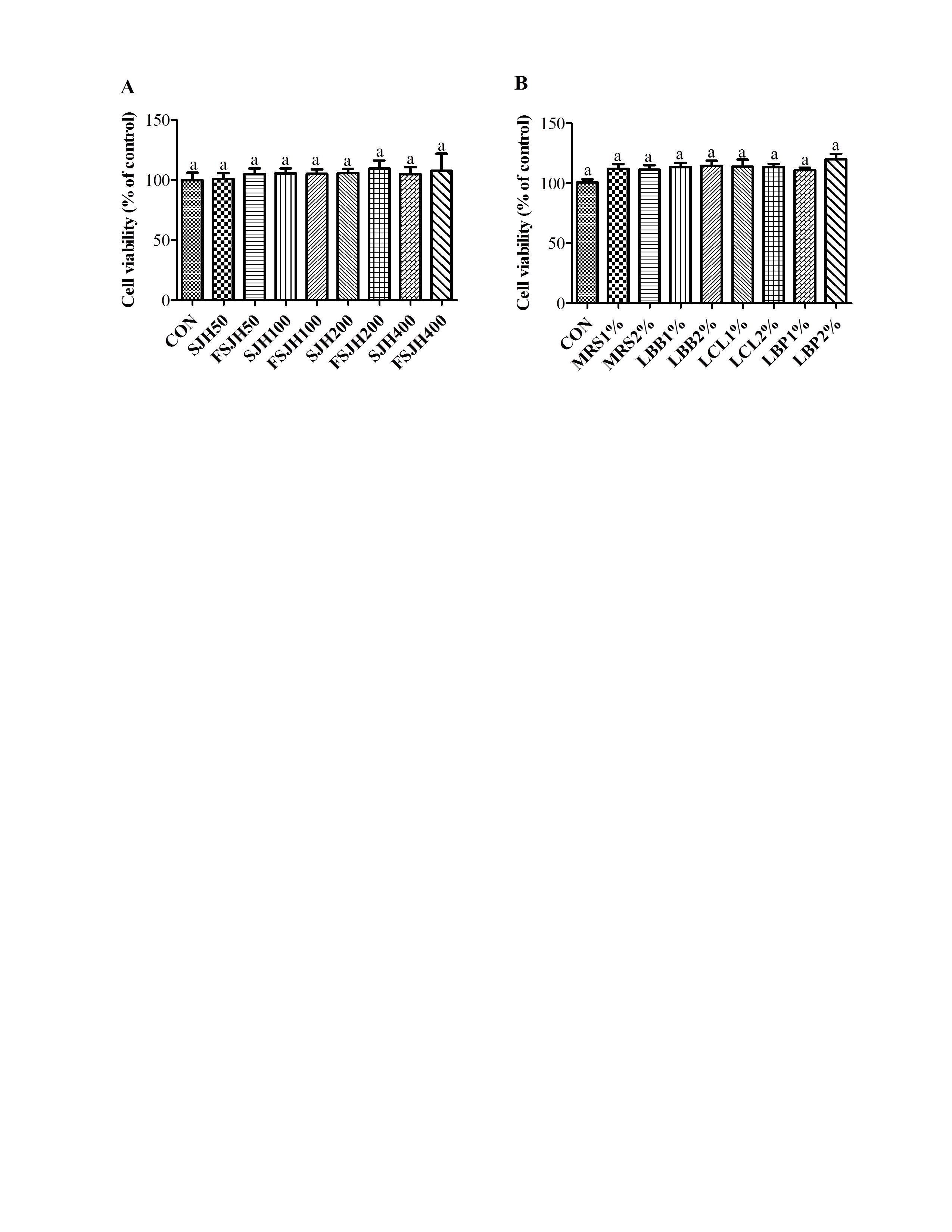

Supplement: FIGURE S2 — Effect of herbal extracts or bacterial strains PMM on cell-viability in HepG2 cells: Cell viability was determined by Ez-cytox assay. The cell viability of HepG2 cells treated with (A) SJH or FSJH extracts (in μg/ml), (B) PMM of bacterial strains (LBB, LCL, or LBP) vs. untreated controls. MRS treatment was considered a positive control in PMM treatment. Data represent the means ± SD (n = 6). Statistical differences between groups were determined by one-way ANOVA. Different letters indicate statistically significant differences between groups, P < 0.05. [file Image_2.jpg]
