# Supplementary material for: A Controlled Fermented Samjunghwan Herbal Formula Ameliorates Non-alcoholic Hepatosteatosis in HepG2 Cells and OLETF Rats
Source: Front Pharmacol. 2018 Jun 19;9:596. doi: 10.3389/fphar.2018.00596 (PMC6018163; doi:10.3389/fphar.2018.00596)
Supplement: TABLE S1 — Sequences of primers used for qPCR. H = Human, R = Rat. [file Table_1.docx]

**SUPPLEMENTARY DATA**

**Table S1: Sequences of primers used for qPCR**

| **Gene** | **Forward** | **Reverse** |
| --- | --- | --- |
| **R-HMGCOR** | **TGCGTGTCCCTGGTCCTA** | **TTGGGTTACTGGGTTTGGT** |
| **R-SREBP** | **AGACTTGGTCATGGGGACAG** | **GGGGAGACATCAGAAGGACA** |
| **R-ACC** | **TACAACGCAGGCATCAGAAG** | **TGTGCTGCAGGAAGATTGAC** |
| **R-AMPK** | **CTATGTGGCTCTGGGCATCTTT** | **ACCACACGCCCTTTCCTATC** |
| **R-LDLR** | **AGGAACTGGCGGCTGAGGAA** | **GCGGCAAATGTGGATCTCG** |
| **R-GAPDH** | **TCCTTGGAGGCCATGTAGGC** | **TGATGACATCAAGAAGGTGGTGAAG** |
| **H-HMGCOR** | **TACCATGTCAGGGGTACGTC** | **CAAGCCTAGAGACATAATCATC** |
| **H-SREBP** | **CAAGATGGTTCCGCCCATCAC** | **CCACTTCATCAAGGCAGACTCG** |
| **H-AMPK** | **AGGATGCCTGAAAAGCTTGA** | **GACAGCCGGAGAAGCAGAAAC** |
| **H-ACC** | **TGTCTGAAGAGATTAGGGAAGT** | **GTTATGTGAAAGATGTGGATGA** |
| **H-LDLR** | **CCCTGCTTGTTTTTCTCTGG** | **TGCAGTTTCCATCAGAGCAC** |
| **H-GAPDH** | **CGCTCTCTGCTCCTCCTGTT** | **CCATGGTGTCTGAGCGATGT** |

**H=Human, R=Rat**
