# Supplementary material for: A Controlled Fermented Samjunghwan Herbal Formula Ameliorates Non-alcoholic Hepatosteatosis in HepG2 Cells and OLETF Rats
Source: Front Pharmacol. 2018 Jun 19;9:596. doi: 10.3389/fphar.2018.00596 (PMC6018163; doi:10.3389/fphar.2018.00596)
Supplement: TABLE S2 — Comparative analysis of herbal extract and postbiotic metabolic media of bacterial strains treatment on FFAs-induced HepG2 cells. ORO, oil red o; TC, total cholesterol; TG, triglycerides. [file Table_2.docx]

**Table S2: Comparative analysis of herbal extract and postbiotic metabolic media of bacterial strains treatment on FFAs-induced HepG2 cells**

| **Item** | **Herbal extracts** | | | | **Postbiotic metabolic media of bacterial strains** | | | | |
| --- | --- | --- | --- | --- | --- | --- | --- | --- | --- |
|  | **CON** | **FFA** | **SJH** | **FSJH** | **CON** | **FFA** | **LBL** | **LBB** | **LBP** |
| **ORO**  **(%)** | 100  (±0) a | 254  (±0) b | 180  (±0) c | 155  (±0) c | 100  (±15) a | 279  (±11) b | 152  (±5) c | 141  (±6) c | 164  (±6) c |
| **TC (%)** | 100  (±29) a | 452  (±84 b | 193  (±40) c | 151  (±57) c | 100  (±23) a | 290  (±28) b | 198  (±5) c | 179  (±13) c | 176  (±6) c |
| **TG (%)** | 100  (±10) a | 283  (±93) b | 157  (±34) c | 128  (±12) c | 100  (±21) a | 402  (±69) b | 282  (±23) c | 287  (±40) c | 263  (±33) c |

**ORO, oil red o; TC, total cholesterol; TG, triglycerides**
